# Idiopathic Intracranial Hypertension as a Differential Diagnosis in Persistent Idiopathic Facial Pain: A Case Report

**DOI:** 10.7759/cureus.63721

**Published:** 2024-07-03

**Authors:** Mohamed Gafar Ahmed, Hourya Alnofaie, Abdullah Aljafar, Hassan Albrahim

**Affiliations:** 1 Oral and Maxillofacial Surgery, King Fahad Hospital of the University, Dammam, SAU; 2 Oral and Maxillofacial Surgery, Princess Nourah Bint Abdulrahman University, Riyadh, SAU; 3 Oral and Maxillofacial Surgery, Saudi Commission for Health Specialties, Riyadh, SAU

**Keywords:** case report, headache, dentofacial pain, neurogenic facial pain, trigeminal neuralgia, idiopathic intracranial hypertension, idiopathic facial pain

## Abstract

Persistent idiopathic facial pain (PIFP), previously known as atypical facial pain (ATFP), is a chronic pain disorder with the characteristic of persistent, undulating pain in the face or the teeth without a known cause or any structural correlation. Women are more commonly affected than men. We report a case of a 38-year-old married female patient with a history of Crohn's disease who presented to the oral and maxillofacial surgery (OMFS) clinic with chronic dull bilateral facial pain and headache mainly affecting the right side of the face and neck without a known cause. She was initially diagnosed with PIFP due to a badly decayed right wisdom tooth. Wisdom teeth were extracted secondary to vague complaints of discomfort due to wisdom teeth; however, no significant improvement was noticed. Further investigations were carried out with new CT scans and magnetic resonance venography (MRV), which revealed evidence of having idiopathic intracranial hypertension (IIH), described as increased intracranial pressure with facial pain, headache, tinnitus, and papilledema. The patient was referred to neurology and received appropriate treatment. She began her treatment with topiramate, then transitioned to acetazolamide, underwent bilateral botulinum toxin (botox) injections into the temporal region, and underwent regular follow-up. The patient was significantly improved. Idiopathic intracranial hypertension must be ruled out in cases of PIFP that do not respond to ordinary treatment measures.

## Introduction

Persistent idiopathic facial pain (PIFP) is a chronic pain disorder [1]. The term was first used in 2004 in the revised version of the International Headache Society classification to replace the term atypical facial pain (ATFP) [2]. Nonetheless, it is a known disorder, first described in 1924 by Frazier as ATFP to differentiate the disorder from trigeminal neuralgia with its classic features of unilateral sharp and severe pain [3]. Persistent idiopathic facial pain is distinguished by its continuous pain of the face and/or teeth with no identifiable neurological deficits, sometimes varying in intensity throughout the day [3]. Although the availability of epidemiological data is limited, women account for 75% to 90% of the cases and are significantly more affected than men [4,5]. The incidence of the first diagnosis is usually between the ages of 30 and 60 [5]. Historically, the disorder was associated with young working women and described as a psychiatric condition with traits of obsession, hysteria, and anxiety [6]. Idiopathic intracranial hypertension (IIH) is a condition of increased intracranial pressure with no clear pathogenesis [7]. Interestingly, IIH is also significantly more common in women [7]. It has a predominant prevalence in women of reproductive age and a striking association with obesity [7]. The classical features of the condition are headaches, the presence of signs of facial pain, and papilledema, as described by Dandy in 1937 [8]. Other associated features of IIH are dizziness, pulsatile tinnitus, and cognitive impairment [9]. There is an enhanced understanding of the radiological signs used to diagnose IIH over the past 20 years, like empty sella turcica [10]. According to the literature, there are no reported cases associating PIFP with IIH. However, three cases were found to relate trigeminal neuralgia to IIH [11,12]. Here, we report, to our knowledge, the first case of IIH, which was initially diagnosed and managed as a case of PIFP.

## Case presentation

**Figure 1:**
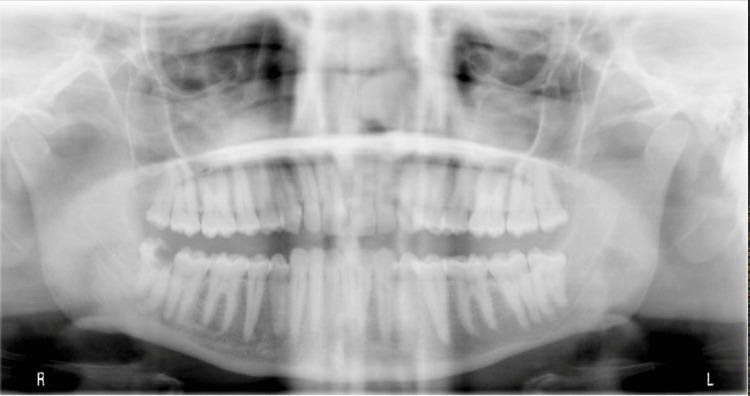
A pre-extraction orthopantomogram of all wisdom teeth

A 38-year-old married female patient with middle socioeconomic status, non-smoker, known case of Crohn’s disease, with insignificant medical family history, presented to the oral and maxillofacial surgery (OMFS) department of King Fahd Hospital of University, Khobar, Saudi Arabia, complaining of facial pain, headache, and tinnitus for a duration of a few months. Bilateral facial pain was continuous, unprovoked, and dull, occasionally accentuating the right side of the face and extending to the neck. The pain intensity was given seven out of 10 using the numerical rating scale of pain. The patient denied any history of facial trauma, infection, or any parafunctional habits. Medical, surgical, and dental histories were unremarkable. All history and data were collected during the first visit for patient assessment and interviewing in the clinic. Upon examination, the patient was vitally stable. There was no clinical lymphadenopathy and no neurological abnormalities. Facial neurosensory testing and facial nerve function were normal. Temporomandibular joint (TMJ) and facial muscle examinations were within normal limits. Intraoral examination showed normal mouth opening, good oral hygiene, multiple restored teeth, and a non-restorable partially impacted tooth #48. An orthopantomogram (OPG) showed normal anatomy and position of TMJ elements, normal maxillary sinuses, multiple restored teeth, and decayed wisdom tooth #48 (Figure 1). 

**Figure 2:**
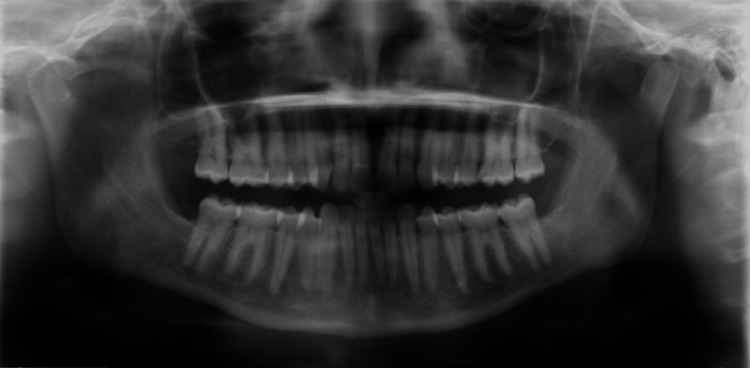
An orthopantomogram performed six months after the extraction of all wisdom teeth

According to the provided history and examination findings, a working diagnosis was elected to be right-sided PIFP, mostly related to decayed tooth number #48, which caused the long-standing pain and evaded the right side of the face. It was then elected to have all wisdom teeth removed along with symptomatic tooth #48 to exclude all possible risk factors and then to keep the patient on regular follow-up visits. Under general anesthesia (GA), the patient underwent dental extraction of all wisdom teeth (#18, #28, #38, and #48). During regular follow-up appointments, wound healing was progressing uneventfully, yet the pain was persistent and continuous most of the day. A new OPG was obtained, and there was no abnormality evident in the patient's anatomy; TMJ, bony aspects, maxillary sinuses, or teeth were all normal. (Figure 2). A more detailed examination was done for the dentoalveolar region, including the TMJ and muscle of mastication, showing no abnormalities. A computed tomography (CT) scan of the brain was requested and showed small hypodensity in the left high parietal region and prominent tortuous bilateral optic nerve sheath complexes with partially empty sella turcica. These findings led us to investigate known IIH. 

**Figure 3:**
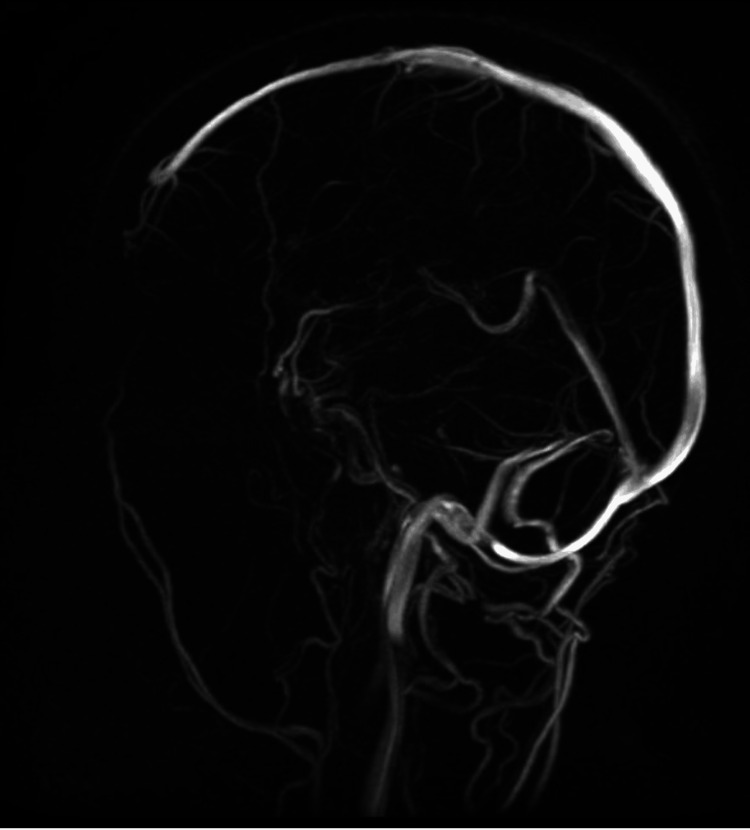
A magnetic resonance venography (sagittal view, T2-weighted) shows stenosis in the left transverse sinus

Magnetic resonance imaging (MRI) and magnetic resonance venography (MRV) were requested to evaluate the cerebellopontine angle to rule out the presence of any neurovascular compression that could be the cause of neurogenic pain. The MRI results showed partially empty sella turcica and prominent tortuous optic nerve sheath complexes, highly suggestive of IIH. Palliative treatment started along with consulting the neurology and ophthalmology departments. An ophthalmology examination revealed papilledema and increased intraocular pressure. Magnetic resonance venography was suggestive of stenosis in the bilateral transverse sinuses, especially on the left side, related to the present IIH (Figure 3).

The patient was diagnosed with IIH and continued her treatment with the neurology department with successful results in pain reduction. They initially started the patient on topiramate 50 mg twice a day (BID) to control her headache symptoms, and she was given acetazolamide (Diamox) 250 mg BID to reduce intraocular pressure. In addition to administering botulinum neurotoxin injections to the bilateral temporal area to alleviate the patient's headache symptoms, which yielded promising results, the patient continued to receive regular follow-up appointments, initially every two weeks, then every month, and finally every three months. Now the patient is being seen every six months on regular appointments, during which the patient has significantly improved and her pain scale has improved to below five out of 10. 

## Discussion

Persistent idiopathic facial pain, which was previously known as ATFP, is described by the International Classification of Headache Disorders as “persistent facial and/or oral pain, with daily recurring variable presentations for more than two hours per day over more than three months, in the absence of clinical neurological deficit” [13]. However, there is no specific mechanism by which facial pain is linked to IIH. On the other hand, trigeminal neuralgia is specific and limited to the distribution of the trigeminal nerve; it is characterized by recurrent unilateral, brief electric shock-like pains, abrupt in onset and termination, limited to the distribution of one or more divisions of the trigeminal nerve, and triggered by innocuous stimuli [13]. One must not confuse different chronic facial pain disorders; the importance of accurate diagnosis for successful therapy is key to managing patients and preventing serious consequences [1]. Due to the location and intensity of pain, many patients might consult a dental healthcare practitioner [13].

In the present case, while the possible source of pain was removed, pain persisted most of the day; hence, it was imperative to accurately diagnose the condition of the patient to improve their quality of life [1]. An initial diagnosis of PIFP was made, but more investigations were needed. The MRI result showed interesting findings that have helped in shifting the diagnosis of the condition to IIH with the highly suggestive feature of partially empty sella turcica [10]. Idiopathic intracranial hypertension is a disorder predominantly in women who are obese and of reproductive age; however, the phenotype of headache is highly variable and may resemble other primary headache disorders [14]. The presence of papilledema in our case was a key feature since clinical presentation is highly variable among patients with signs of IIH [8,14]. Symptoms of IIH may vary, including headaches that may mimic migraine or tension headaches, dizziness, pulsatile tinnitus, visual loss, unilateral or bilateral visual obscuration, horizontal diplopia, and cognitive impairment, all of which were ruled out in our patient [14].

In the literature, there are few articles associating IIH with trigeminal neuralgia; however, there was no association between PIFP and IIH, which acts as a limitation in our study [15-18]. The pain described in the other articles was unilateral; however, the presentation differs [11,12]. The pain in the current case, in comparison to the previous cases, was bilateral in origin, with symptoms of intermittent migraine episodes but mostly affecting the right side of the face. Iftikhar et al. reported a case of IIH resembling symptoms of sinusitis [11]. Failure to address the medical condition of patients suffering might cause enormous dilemmas, including poor quality of life, persistent pain, and prolonged duration of treatment [11].

Since signs and symptoms of these disorders are not consistent, a patient might seek advice from multiple specialties, including dental, otorhinolaryngology, and psychology healthcare providers, before approaching neurology [11]. The lack of a comprehensive evaluation of the cervical spine represents an additional limitation in our diagnostic process. Including this assessment could potentially alter the differential diagnosis or influence the management plan. For instance, if cervical pathology had been identified, which leads to headaches or trigeminal neuralgia [19], treatment strategies might have included physical therapy, cervical spine manipulation, or targeted nerve blocks [19]. After going through a long-standing experience with an unknown source of facial pain and not improving with pain medications, our patient was finally diagnosed correctly and managed appropriately based on her positive results and feedback since her last follow-up visit. Collecting all the necessary data, considering all potential differential diagnoses related to symptoms of an unknown source of chronic facial pain, and involving the appropriate teams and specialties using the latest advanced technologies as a diagnostic aid could significantly reduce the time it takes for both physicians and patients to treat uncommon diseases.

## Conclusions

Accurate diagnosis is of the utmost value for a patient's quality of life. The inconsistency of signs and undesirable symptoms associated with headache disorders might cause the patient to consult different healthcare providers; hence, familiarity with these disorders is imperative. Misdiagnosis of any condition may cause physical and psychological fatigue, along with financial burden and a huge decrease in quality of life. Interestingly, no literature from oral and maxillofacial surgery, pathology, or medicine has linked PIFP with IIH, although some clinical features are quite similar. Reporting this as likely to be the first case of association between PIFP and IIH may highlight some of the challenges patients and healthcare providers may face and draw attention to the significant number of similar cases that could have been misdiagnosed. The role of maxillofacial surgeons and dental healthcare providers in diagnosing these conditions and directing the patients to treatment will prevent further severe complications.
